# Predictors of changes after reasoning training in healthy adults

**DOI:** 10.1002/brb3.1861

**Published:** 2020-09-27

**Authors:** Mandy Roheger, Elke Kalbe, Anne Corbett, Helen Brooker, Clive Ballard

**Affiliations:** ^1^ Department of Medical Psychology Neuropsychology and Gender Studies & Center for Neuropsychological Diagnostics and Intervention (CeNDI) Faculty of Medicine and University Hospital Cologne Cologne Germany; ^2^ Department of Neurology University Medicine Greifswald Greifswald Germany; ^3^ Institute of Health Research University of Exeter Medical School University of Exeter Exeter UK

**Keywords:** healthy older adults, prediction, reasoning cognitive training

## Abstract

**Objectives:**

To investigate predictors of performance changes and their time course in healthy older adults.

**Design:**

A post hoc analysis of a RCT investigating the effect of reasoning cognitive training (ReaCT) compared to an active control group (CG) during a time course.

**Setting and participants:**

An online, home‐based RCT including *n* = 4,310 healthy participants (ReaCT: *n* = 2,557; CG: *n* = 1,753) aged 50 years and older.

**Methods:**

Multiple regression analyses were conducted to investigate predictors (age, sex, education, severity of depression, number of training sessions the participants attended, and neuropsychological baseline values) of the outcome measures grammatical reasoning, working memory, digit vigilance, verbal short‐term memory, and verbal learning at 6 weeks, 3, and 6 months.

**Results:**

Being female and lower education predicted improvements in grammatical reasoning scores at 6 weeks and 3 months of training.

**Conclusion and implication:**

Identifying predictors for nonpharmacological interventions may help to set up a personalized medicine approach in order to prevent cognitive decline in healthy older adults.

## Graphical abstract

We investigated which variables predict changes after an online reasoning cognitive training program and could show that being female and lower education predicted improvements in grammatical reasoning scores at 6 weeks and 3 months of training. Identifying predictors for nonpharmacological interventions may help to set up a personalized medicine approach in order to prevent cognitive decline in health older adults.

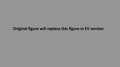

## BRIEF SUMMARY

Identifying predictors of changes after nonpharmacological interventions is essential for a personalized medicine approach in order to prevent cognitive decline. Here, being female and lower education predicted improvements at 6 weeks and 3 months of a reasoning training.

## 1 INTRODUCTION

While the efficacy of cognitive trainings (CT) in healthy older adults to improve cognitive outcomes is shown in several systematic reviews and meta‐analyses (e.g., Chiu et al., 2017; Kelly et al., 2014; Martin et al., 2011), one particular question that remains underinvestigated is as follows: Which specific individual characteristics (e.g., sociodemographic variables, (neuro‐)psychological variables, genetic and brain imaging parameters) predict changes in cognitive outcomes after a specific CT? Therefore, with the help of regression analyses, we want to investigate in a randomized paradigm including reasoning cognitive training (ReaCT) and a “no training” control group the following research question: Which variables predict change of cognitive function after a ReaCT?

## 2 METHODS

### 2.1 Study design

Data were taken from a double‐blind 6‐month online randomized three‐arm controlled trial (general cognitive training [GCT] versus ReaCT versus active control group [CG]) with healthy older adults. In previous papers, short‐ and long‐term effects of this randomized controlled trial (RCT) were reported (Corbett et al., 2015), as well as predictors of cognitive change in the GCT group (Roheger et al., 2020). Results of the short‐ and long‐term effects of this RCT indicated that the ReaCT significantly benefits to grammatical reasoning, verbal short‐term memory, spatial working memory, and verbal learning at 6 months in comparison with controls. Significant benefit was further seen at 3 months in the domain reasoning for ReaCT (Corbett et al., 2015). Results of predictors of cognitive change in the GCT group showed that being female was predictive for improvement in grammatical reasoning at 6 weeks, and lower cognitive baseline scores were predictive for improvement in spatial working memory and verbal learning at 6 months (Roheger et al., 2020).

In the present study, only data from the ReaCT and the CG were used with four measurement times at baseline, 6 weeks, 3 months, and 6 months. The present study follows an exploratory approach with the aim to identify possible predictors of the outcomes investigated in the original study (grammatical reasoning, spatial working memory, digit vigilance, verbal short‐term memory, verbal learning), as results regarding predictors of ReaCT are rare so far and a systematic overview is lacking. Therefore, we did not state any a‐priori hypothesis. The study was approved by the St Thomas' Hospital Research Ethics Committee (Ref: 09/H0802/85) and registered on the International Standard Randomized Controlled Trial Number (ISRCTN) clinical trial database (Ref: ISRCTN72895114).

### 2.2 Participants

Eligibility criteria were as follows: (1) individuals older than 50 years and (2) access to a computer and the internet. Adults older than 50 years in the United Kingdom and internationally were invited to take part in this online RCT due to a partnership with the British Broadcasting Corporation (BBC), Alzheimer's Society (UK), and the Medical Research Council. Individuals could register and consent to the study through a secure connection and ethically approved online process. After that, participants received their own login details and were randomized to a study group (GCT, ReaCT, or CG). Reminder emails throughout the intervention tried to ensure that participants continue their training and complete their online cognitive assessments. A summary of performance and reinforcing text were automatically generated at the end of training sessions. Participants were included in the study when they participated in at least one training session.

### 2.3 Reasoning cognitive training

The ReaCT consisted of six cognitive tasks that trained reasoning and planning (3 tasks for each outcome, for an overview, see Table 1). Participants were recommended to train for at least 10 min per day; yet, flexibility in the training duration was allowed. Task difficulty increased as participants improved so as to maintain the challenge and maximize performance. Participants could choose which sessions they would like to train and in which order. The CG performed online tasks involving a game in which people were asked to put a series of statements in the correct numerical order. In each session, the CG were asked five obscure knowledge questions (e.g., what year did Henry VIII die?) from one of six general categories (population, history, duration, pop music, miscellaneous numbers, and distance) and were asked to place answers in correct chronological order using any available online resource.

**Table 1:** Training sessions included in the reasoning cognitive training Packages

Training session	Task	Main outcome measure
Reasoning 1	Use weight relationships, implied by the position of two seesaws with objects at each end, to select the heaviest object from a choice of three.	Total number of correct trials across the two runs.
Reasoning 2	Select the “odd one out” from four shapes that varied in terms of color, shape, and solidity (filled/unfilled).	Total number of correct trials across the two runs.
Reasoning 3	Move crates from a pile, each move being made with reference to the effect that it would have on the overall pattern of crates and how the result would affect future moves.	Total number of correct trials across the two runs.
Planning 1	Draw a single continuous line around a grid, planning ahead such that current moves did not hinder later moves.	Number of problems completed in three minutes.
Planning 2	Move objects around between 3 jars until their positions matched a “goal” arrangement of objects in 3 reference jars.	Total number of correct trials across the two runs.
Planning 3	Slide numbered “tiles” around on a grid to arrange them into the correct numerical order.	Number of problems completed in 3 min.

Note

This table was taken and modified from Corbett et al. (2015). All sessions consisted of two 90 s “runs.”

### 2.4 Outcome measures

In the present study, we investigated the following outcome: grammatical reasoning, spatial working memory, digit vigilance, verbal short‐term memory, and verbal learning. All investigated cognitive outcome measures were completed at baseline (after registering for the trial and before starting the first training session; T1), at 6 weeks (T2), 3 months (T3), and 6 months (T4) for the ReaCT and the CG. Data were collected from all participants irrespective of the number of completed training sessions and were adapted for online use from available validated cognitive assessment tools. *Grammatical reasoning* was measured with the Baddeley Grammatical Reasoning Test (Baddeley, 1968), using the total number of trials answered correctly in 90 s minus the number answered incorrectly as the outcome variable. *Spatial working memory* was measured with the Spatial Working Memory test (Owen et al., 1990). In this test, participants are asked to search a series of on‐screen boxes to find a hidden symbol. The main outcome was the change in the score of the average number of boxes in the successfully completed trials. A version of the “digit span” task in which each successful trial is followed by a digit span that is one digit longer than the last one, and each unsuccessful trial is followed by a digit span one digit shorter than the last, was used to measure *digit vigilance*. The average number of digits in all successfully completed trials was used as the main outcome. *Verbal short‐term memory* was measured using the paired associates test (Owen et al., 1993; please note that the paired associate test is often declared to measure episodic memory; however, the authors decided to stay consistent with previous publication of the data for the naming of the outcome, as the test also has a verbal memory component). Participants are asked to select the correct location of different objects in “windows,” which they had previously been shown. The average number of completed correct object–place associations in the trials was used as the main outcome measure. The revised Hopkin's Verbal Learning test (Benedict et al., 1998) was used to measure *verbal learning*. The test includes six different forms, each containing 12 nouns and four words, which are taken each from one of three semantic categories to be learned over the course of three learning trials. The learning trials are followed by a recognition trial 20–25 min later composed of 24 words, including the 12 target words and 12 false positives.

### 2.5 Predictors

The predictors age, sex, education, group, severity of depression, neuropsychological baseline values, and number of intervention sessions were assessed. Age (numerical variable, in years), sex (assessed as a binary variable: male versus female), education (categorized in five categories: none, primary school, secondary school, further education, university graduate), and severity of depression (assessed as a numerical variable on the Patient Health Questionnaire (Kroenke et al., 2001)) and neuropsychological baseline values were assessed before the training started. The Patient Health Questionnaire is a multiple‐choice self‐report inventory that is used as a screening tool for mental health disorders. The neuropsychological baseline values (assessed at pretest) were matched to the outcome (e.g., grammatical reasoning baseline value was used in the regressions that used grammatical reasoning as the outcome value). The predictor “group” was dichotomized (ReaCT versus CG). The number of training sessions was assessed as the total number of training sessions a participant completed until the time of measurement (as a continuous variable). Predictor assessment was blinded due to the online study design.

### 2.6 Statistical analyses

All analyses were performed using the Statistic software R (R Core Team 2014), and all significance levels were set at *α* = 0.05. Descriptive statistics are displayed with means and standard deviations for numerical variables, and all other values are displayed in *n* (%) and were calculated using *t* tests or chi‐square tests, where appropriate.

We calculated predictions of changes after reasoning training in the ReaCT group at three different time points: 6 weeks (T2), 3 months (T3), and 6 months (T4). Multiple regressions were calculated using the change scores (T2 ‐ T1; T3 ‐ T1; T4 ‐ T1) of grammatical reasoning, spatial working memory, digit vigilance, paired associative learning, and verbal recall as dependent variables. The following predictors were integrated: baseline score of dependent variable (T1), group (ReaCT and CG), age, sex, education, severity of depression, number of training sessions, and all interactions between all predictors with the group. Integrating T1 in the regression controls for differences in the variable of interest that were present prior to the intervention, similar to a covariate in an analysis of covariance. A recent simulation study shows that models including the pretest score as a predictor yield a better power to unveil a significant main effect of the predictor of interest or the interaction effect between the group and predictor of interest than models that do not include the pretest score as a predictor (Mattes & Roheger, 2020). Effect sizes are displayed in the beta weights of the regression. *ß* > 0.1 indicates a small effect, *ß* > 0.3 indicates a medium effect, and *ß* > 0.5 indicates a large effect (Cohen, 1992). We are particularly interested in the results of the interaction terms (group*predictors), because these indicate significant predictors only for the ReaCT group compared with the CG. We used the Bonferroni correction to account for multiple testing at each time point (*p* = .05/5 outcomes at one measurement point). All multiple regression analyses were also conducted only with the sample size of the 6‐month sample (*n* = 595) for sensitivity analysis.

## 3 RESULTS

### 3.1 Demographic characteristics of the sample at all three measurements

**Figure 1:**
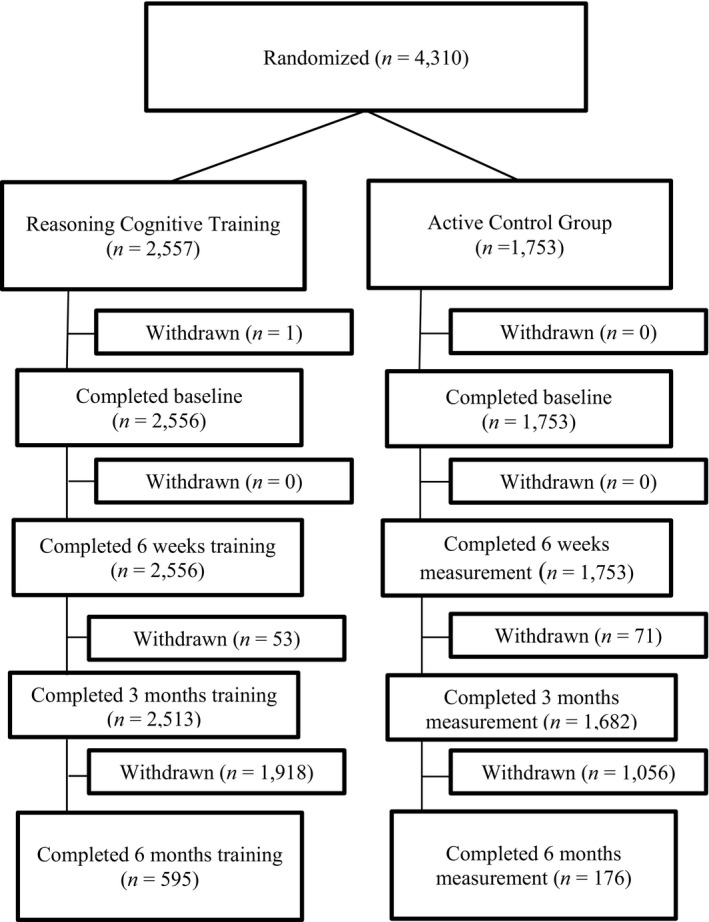
Overview of flow of participants throughout the study

A total of *n* = 2,556 participants were included in the ReaCT group at baseline, and *n* = 1,753 participants were included in the CG at baseline. The different numbers of participants between ReaCT and CG are caused by the fact that participants were only included in the study after randomization if they did at least one session of the intervention (either ReaCT or CG). A flowchart of the participants throughout the study is displayed in Figure 1. Table 2 presents the demographic characteristics of the ReaCT group and the CG at baseline, 6 weeks, 3 months, and 6 months. Table 3 demonstrates the differences between those participants who completed the training at 6 months of follow‐up and those who dropped out of the study. In the ReaCT, participants who dropped out of the study were younger and differed in their educational level; participants in the CG who dropped out of the study were also younger and had higher scores in their performance on the paired associate learning test.

**Table 2:** Descriptive statistics of the Reasoning Cognitive Training Group and the active Control Group at baseline, 6‐week follow‐up, and at 3‐ and 6‐month follow‐up

Characteristics	Participants who completed baseline and 6‐week follow‐up (*n* = 4,309)			Participants who completed 3‐month follow‐up (*n* = 4,195)			Participants who completed 6‐month follow‐up (*n* = 771)		
	ReaCT *n* = 2,556	Control *n* = 1753	*p*‐value	ReaCT *n* = 2,513	Control *n* = 1,682	*p*‐value	ReaCT *n* = 595	Control *n* = 176	*p*‐value
Age,y	58.5 (6.5)	59.1 (6.6)	.002	58.2 (6.5)	59.1 (6.6)	.005	59.03 (6.44)	60.81 (7.24)	.002
Sex, female	1752 (68.5)	1,093 (62.4)	.000	1,720 (68.7)	1,036 (68.8)	.000	425 (71.4)	98 (55.7)	.000
Ethnic origin			.113			.133			.721
Asian	25 (1)	10 (0.6)		23 (0.9)	9 (0.5)		8 (1.3)	1 (0.6)	
Black	7 (0.3)	4 (0.2)		7 (0.3)	4 (0.2)		2 (0.3)	1 (0.6)	
Middle Eastern	2 (0.1)	4 (0.2)		2 (0.1)	3 (0.2)		0 (0.0)	0 (0.0)	
Mixed White/Black	8 (0.3)	8 (0.5)		8 (0.3)	8 (0.5)		1 (0.1)	0 (0.0)	
Mixed White/Asian	9 (0.4)	11 (0.6)		8 (0.3)	11 (0.7)		1 (0.2)	1 (0.6)	
White	2,478 (96.9)	1707 (97.4)		2,427 (97.0)	1628 (97.4)		582 (97.8)	172 (97.7)	
Other	28 (1.1)	9 (0.5)		28 (1.1)	9 (0.5)		1 (0.2)	1 (0.6)	
Education			.049			.094			.039
None	44 (1.7)	37 (2.1)		41 (1.6)	34 (2.0)		5 (0.8)	2 (1.1)	
Primary school	14 (0.6)	9 (0.5)		13 (0.5)	9 (0.5)		3 (0.5)	2 (1.1)	
Secondary school	400 (15.6)	320 (18.3)		391 (15.6)	297 (17.8)		86 (14.5)	37 (21.0)	
Further education	777 (30.4)	556 (31.7)		759 (30.3)	531 (31.8)		175 (29.4)	61 (34.7)	
University graduate	1,322 (51.7)	831 (47.4)		1,299 (51.9)	801 (47.9)		326 (54.8)	74 (42.0)	
Baddeley Grammatic Reasoning Test	14.4 (5.3)	14.1 (5.3)	.134	14.4 (5.2)	14.2 (5.2)	.131	14.57 (5.28)	13.71 (5.24)	.057
SWM Test	5.0 (1.2)	5.0 (1.2)	.110	5.0 (1.2)	4.9 (1.2)	.103	5.06 (1.26)	4.91 (1.22)	.174
Paired Associate Learning Test	3.5 (0.6)	3.5 (0.6)	.102	3.5 (0.6)	3.5 (0.6)	.130	3.53 (0.61)	3.42 (0.60)	.017
Digit Span Ladder Test	4.8 (1.1)	4.7 (1.1)	.559	4.8 (1.1)	4.7 (1.1)	.508	4.85 (1.11)	4.63 (1.11)	.031

Note

Age (in years), Baddeley Grammatic Reasoning Test, SWM Test, Paired Associate Learning Test, and Digit Span Ladder Test are reported with means and standard deviations. All other values are *n* (%). P‐values indicate group differences between the two groups at each of the three time points. Group differences were calculated using *t* tests and chi‐square tests, where appropriate.

Abbreviations: ReaCT, reasoning cognitive training; SWM, Spatial working memory.

**Table 3:** Descriptive statistics of attendants and dropouts throughout the study at 6‐month follow‐up

	ReaCT			Control		
	Responder (*n* = 595)	Dropout (*n* = 1,962)	*p*‐value	Responder (*n* = 176)	Dropout (*n* = 1,577)	*p*‐value
Age, year	59.03 (6.44)	58.35 (6.55)	.025	60.81 (7.24)	58.96 (6.51)	.000
Sex, female	425 (16.6)	1,327 (51.90)	.081	98 (5.60)	995 (56.8)	.054
Ethnic origin						
Asian	8 (0.3)	17 (0.70)	<.05	1 (0.10)	9 (0.5)	<.05
Black	2 (0.1)	5 (0.20)	<.05	1 (0.10)	3 (0.2)	<.05
Middle Eastern	0 (0.0)	2 (0.10)	<.05	0 (0.00)	4 (0.2)	<.05
Mixed White/Black	1 (0.0)	7 (0.30)	<.05	0 (0.00)	8 (0.5)	<.05
Mixed White/Asian	1 (0.0)	8 (0.30)	<.05	1 (0.10)	10 (0.6)	<.05
White	582 (22.8)	1896 (74.10)	<.05	172 (9.80)	1535 (87.6)	<.05
Other	1 (0.0)	27 (1.10)	<.05	1 (0.10)	8 (0.5)	<.05
Education						
None	5 (0.2)	39 (1.50)	<.05	2 (0.10)	35 (2.2)	<.05
Primary school	3 (0.1)	11 (0.40)	<.05	2 (0.10)	7 (0.4)	<.05
Secondary school	86 (3.4)	314 (12.30)	<.05	37 (2.10)	283 (16.1)	<.05
Further education	175 (6.8)	602 (23.50)	<.05	61 (3.50)	495 (28.2)	<.05
University graduate	326 (12.7)	996 (39.00)	<.05	74 (4.20)	757 (43.2)	<.05
Baddeley Grammatic Reasoning Test	14.57 (5.28)	14.33 (5.25)	.331	13.71 (5.24)	14.19 (5.27)	.250
SWM Test	5.06 (1.26)	5.00 (1.23)	.310	4.91 (1.22)	4.96 (1.19)	.649
Paired Associate Learning Test	3.53 (0.61)	3.53 (0.60)	.993	3.42 (0.60)	3.53 (0.58)	.014
Digit Span Ladder Test	4.85 (1.11)	4.74 (1.07)	.019	4.63 (1.11)	4.72 (1.11)	.300

Note

Age (in years), Baddeley Grammatic Reasoning Test, SWM Test; Paired Associate Learning Test, and Digit Span Ladder Test are reported with means and standard deviations. All other values are *n* (%).

Abbreviations: GCT, general cognitive training; ReaCT, reasoning cognitive training; SWM, spatial working memory.

### 3.2 Predictors of changes after reasoning training at all three measurements

An overview of the results of the prediction analyses of all three time points (6 weeks, 3 months, and 6 months) is provided in Table 4. At 6‐week measurement (T2), results showed that for grammatical reasoning, higher scores in the ReaCT group were predicted by female sex (*ß* = 0.45), indicating a medium effect, and lower education (*ß* = 0.13), indicating a small effect. At 3‐month measurement (T3), again higher scores in the domain grammatical reasoning were predicted by female sex (*ß* = 0.47), indicating a medium effect, and lower education (*ß* = 0.15), indicating a small effect. No significant interaction terms were seen when investigating the other outcomes, which had an effect size of *ß* ≥ 0.1. Also, results showed no significant interaction terms for any of the investigated dependent variables at 6‐month measurement (T4).

**Table 4:** Predictors of reasoning cognitive training success at 6‐week, 3‐month, and 6‐month follow‐up

Predictors	6 weeks (*n* = 4,309) Dependent variables:					3 months (*n* = 4,195) Dependent variables:					6 months (*n* = 771) Dependent variables:				
	GR	SWM	DV	PAL	VL	GR	SWM	DV	PAL	VL	GR	SWM	DV	PAL	VL
Intercept	12.37***	3.90***	3.87***	3.44***	6.12***	12.47***	3.90***	3.98***	3.74***	0.67	13.18***	4.59***	4.04***	3.44***	2.27
Group	−0.54	0.07	0.43	0.16	−0.21	0.32	0.07	0.40	0.08	0.76	2.75	1.03	1.83*	0.40	2.81
Time 1	−0.48***	−0.65***	−0.58***	−0.74***	−1.11***	−0.48***	−0.65***	−0.62***	−0.73***	−1.00***	−0.48***	−0.56***	−0.63***	−0.79***	−0.80***
Age	−0.07***	−0.00	−0.01**	−0.01***	−0.03+	−0.07***	−0.00	−0.01*	−0.02***	0.00	−0.07+	−0.02+	−0.01	0.01	0.01
Sex	−0.18	−0.09	−0.15***	0.03	0.26+	0.06	−0.09	−0.08+	0.02	0.26+	0.13	−0.29+	−0.10	0.05	−0.20
Education	−0.28***	−0.03*	−0.04**	−0.02*	−0.00	−0.26***	−0.03*	−0.04**	−0.00	0.07+	−0.29+	−0.03	−0.03	−0.03	0.03
Depression	−0.00+	0.00	−0.00	−0.00	0.00	−0.00*	0.00	−0.00	−0.02***	0.02	0.03	0.02	−0.02	−0.02	0.03
Number of training sessions	0.00*	−0.00	0.00	−0.00	−0.00+	0.00**	−0.00	−0.00	−0.00	−0.00	0.00	−0.00	0.00	0.01	0.00
Time 1*Group	0.01	−0.00	−0.06*	−0.00	0.12	0.01	−0.00	−0.00	−0.02	0.01	−0.06	−0.17*	−0.09	0.04	−0.20
Age * Group	0.02	−0.00	−0.00	−0.00	0.00	0.01	−0.00	−0.00	−0.00	−0.01	−0.01	−0.00	−0.02	−0.00	−0.04
Sex * Group	0.45+	0.07	0.05	−0.03	−0.00	0.47+	0.07	0.00	0.02	0.07	−0.03	0.08	0.06	−0.10	0.44
Education * Group	0.13+	0.01	0.02	0.01	−0.09+	0.15*	0.01	0.03*	−0.00	−0.06	0.25	0.02	0.03	0.02	−0.03
Depression*Group	0.03	−0.00	−0.00+	−0.01**	−0.03	0.00	−0.00	−0.01*	0.02*	−0.06*	−0.02	−0.02	−0.00	−0.00	−0.06
Number of training sessions * Group	−0.00	0.00*	0.00	0.00	0.00*	−0.00	0.00*	0.00+	0.00	0.00	0.00	0.00	−0.00+	−0.00	−0.00
Adjusted *R* ^2^	0.27***	0.31***	0.29***	0.37***	0.29***	0.27***	0.32***	0.31***	0.36***	0.44***	0.33***	0.43***	0.38***	0.38***	0.35***

Note

All participants older than 50 years were included (reasoning cognitive training and active control group). Significant codes: *** = 0.001; ** = 0.01; * = 0.05, "+" = 0.1. Reference groups: control group, male sex, and university graduate.

Abbreviations: DV, digit vigilance; GR, grammatical reasoning; PAL, paired associate learning; SWM, spatial working memory; VL, verbal learning.

## 4 DISCUSSION

The present paper investigated possible predictors for changes after ReaCT in healthy older participants over a time course of 6 weeks, 3 months, and 6 months. The main results are that (i) being female is predictive for improvements in grammatical reasoning test performances at 6 weeks and 3 months of training showing a medium effect, (ii) that lower education is predictive for improvement in grammatical reasoning tests at 6 weeks and 3 months, showing a small effect, and (iii) that other outcome variables were not predicted by any of the included parameters.

Meta‐analytic data on sex differences in cognition demonstrate that healthy women (aged 67–89) perform better than men on tests of verbal learning and memory (Munro et al., 2012) and women also outperform men in syntactic complexity and grammatical diversity (Moscoso Del Prado Martín, 2017), pointing to stronger cognitive plasticity for verbal tasks in women (Beinhoff et al., 2008). Possibly, this advantage could explain our results that being female is predictive for improvements in grammatical reasoning test performance so that women are capable to improve faster in verbal tasks then men. Even though data on sex difference are still rare in the field of prediction of changes after nonpharmacological trainings, we were also able to show in our analysis on the GCT data taken from the same RCT as the present data (Corbett et al., 2015) that being female was a predictor for changes in grammatical reasoning after 6 weeks (Roheger et al., 2020). Summarized, our present results serve as a cross‐validation for our previous results and support the notion of a “sex‐specific plasticity.”

Lower education was predictive for improvements in grammatical reasoning test performance at 6 weeks and 3 months of training. This result is in line with further studies investigating predictors of changes after cognitive training (Roheger et al., 2019) and memory training (McDougall et al., 2010; Park et al., 2018) in healthy older adults for several cognitive domains. Education may not only be a proxy variable for socioeconomic status, early life factors, occupational health, or even the willingness to engage in lifelong learning or new activities (Krieger et al., 1997), it is also often used as a proxy of cognitive reserve (Rouillard et al., 2017). Differential educational environments which are experienced during lifetime may affect individuals’ cognitive reserve and therefore may explain differences in cognitive outcomes in later life (Mantri et al., 2019). Educational opportunities in early life may have the potential to potentiate new neural networks (Marques et al., 2016) and allow individuals to develop different compensatory strategies (Mantri et al., 2019). As a result, participants with high education typically already function at a good level, whereas participants with lower education can benefit from ReaCT by developing and learning new strategies and tasks (in literature often referred to as the “compensation account” (Lövdén et al., 2012)). Further, results of longitudinal studies in the field have reported a steeper cognitive decline for higher educated adults, again showing more room for improvement in these participants (Salthouse, 2019).

Results showed that lower education and being female only significantly predict improvements in ReaCT at 6 weeks and 3 months, but not at 6 months, indicating a time course within the data (which we also found in our analysis on the GCT data (Roheger et al.,^2020^)). Regarding sex differences over the time course, a possible explanation might be that women could be more capable than men of activating their former resources in verbal domains immediately at the beginning of the training (Beinhoff et al., 2008). Verbal resources seem stronger in women and enable a faster activation of knowledge and strategies in the specific grammatical reasoning task. However, future research has to elaborate more on this specific topic. Regarding educational differences, the same pattern occurs: Lower education is predictive for improvements at 6 weeks and 3 months, but not at 6 months. A possible explanation could be that after 6 months of training, also participants with initially lower educational status perform on an upper level and thereby reduce the differences to participants with initially higher educational status as they had enough time to improve their performance. This hypothesis needs more research, especially as a comparison of our study with other training studies is difficult because our data refer to an ongoing training, whereas most other studies have a specific training duration and predictors of improvements after training then refer to postintervention or follow‐up measurements without intercalated training intervals.

Strengths of the present paper are that it investigates predictors of improvements of a ReaCT over the time course in a large sample taken from an online RCT, which has rarely been conducted. As a possible limitation, the sample may be biased, however, due to the fact that often highly motivated and highly educated participants conduct CT studies per se (Schubert et al., 2014), and that this may have influenced our results. Further, we could not ensure that participants are not in contact with each other and shared information about the different groups they participated in. However, as participants did not have any contact details of other participants, this may only be the case when sharing information with, for example, participating people living in the same household and/or friends, and therefore, the risk was rated as rather low by the study authors. Further, there was a high dropout in the CG at 6‐month measurement, which is the reason we also calculated sensitivity analyses to check our results. This high dropout rate at 6‐month measurement might be due to a lack of motivation of the participants possibly caused by the fact that the trained tasks were repetitive. This might also explain the fact that younger participants were more likely to drop out of the study; it may be possible that the task was not difficult enough or younger participants were more easily bored by the repetitive structure of the training. Future studies should include further variables of interests as possible predictors, for example, overall cognitive performance at baseline, as well as the total time of training. As a final limitation, participants were not screened for dementia before participating in the study.

## 5 CONCLUSION AND IMPLICATIONS

In conclusion, our study showed that sociodemographic variables (namely sex and education) may predict improvements after ReaCT in healthy older adults with medium and low effect sizes, respectively, and there seems to be a differential time course for this prediction. Further research should focus on unraveling prediction patterns and underlying mechanisms for improvements after nonpharmacological interventions, as these patterns might help to tailor nonpharmacological interventions to individuals with different profiles. By identifying different subgroups within the general population of healthy older adults, nonpharmacological interventions can then be designed to fit their specific needs (e.g., more difficult cognitive trainings for participants with a higher educational level to avoid ceiling effects and also give these individuals an opportunity to improve). The ultimate goal is then to optimize the prevention of cognitive decline in a personalized medicine approach.

## CONFLICTS OF INTEREST

The authors declare no conflict of interests.

## ACKNOWLEDGEMENTS

Open access funding enabled and organized by Projekt DEAL.

## AUTHOR CONTRIBUTIONS

All authors meet the criteria for authorship stated in the Uniform Requirements for Manuscripts Submitted to Biomedical Journals, and all authors' specific areas of contributions are listed below:

All authors contributed to study concept and design.

AC, HB, and CB involved in acquisition of data.

MR, EK, and CB analyzed and interpreted the data.

MR drafted the manuscript.

AC, HB, CB, and EK involved in critical revision of the manuscript for important intellectual content.

### Peer Review

The peer review history for this article is available at https://publons.com/publon/10.1002/brb3.1861.

## Data Availability

Data are available and shared on reasonable request.
